# Postoperative complications analysis of circular stapled versus linear stapled anastomosis for patients undergoing esophagectomy: a systematic review and meta-analysis

**DOI:** 10.1186/s13019-023-02309-y

**Published:** 2023-08-09

**Authors:** Hao-Yu Gu, Jing Luo, Yong Qiang

**Affiliations:** 1 Medical College, Nantong University, Nantong City, Nantong, 226000 Jiangsu P.R. China; 2 Department of Cardiothoracic Surgery, Nanjing Jinling Hospital, East Region Military Command General Hospital, Nanjing, 210000 Jiangsu P.R. China

**Keywords:** Anastomotic leakage, Anastomotic stricture, Circular, Linear, Anastomosis

## Abstract

**Background:**

The choice of anastomosis technique after esophagectomy is closely associated with the postoperative complications. Whether circular stapled or linear stapled anastomosis is the optimal technique has not been established. Therefore, we conducted this meta-analysis to show the latest and most comprehensive published assessment of circular stapled anastomosis in comparison with linear stapled anastomosis in postoperative complications.

**Methods:**

Databases (PubMed, Embase, Web of science, Cochrane Library) were searched for all randomized controlled trials and comparative studies comparing circular stapled anastomosis with linear stapled anastomosis after esophagectomy. The odd ratio and mean difference with 95% confidence interval were calculated. We used the Higgins I² statistics to assess the statistical heterogeneity between studies. Review manager (version 5.4) software was used in this analysis.

**Results:**

Sixteen studies with 2322 patients were included in our study. The study demonstrated that the use of linear stapled technique after esophagectomy could reduce the risk of both anastomotic leakage (P = 0.0003) and stricture (P < 0.00001) compared with circular stapled technique. Stratification by anastomotic site showed that no matter what kind of anastomotic site (cervical or thoracic anastomosis) was used, linear stapled anastomosis could effectively reduce the anastomotic stricture in comparison with circular stapled anastomosis. Moreover, linear stapled anastomosis could decrease the risk of thoracic anastomotic leakage. There were no significant differences between circle stapled anastomosis and linear stapled anastomosis in reflux esophagitis (P = 0.17), pneumonia (P = 0.91), operation time (P = 0.41) and hospital stay (P = 0.38).

**Conclusions:**

The study suggested that linear stapled anastomosis could be considered to be an optimal treatment associated with a reduced risk of anastomotic leakage and stricture in comparison with circular stapled anastomosis.

**Supplementary Information:**

The online version contains supplementary material available at 10.1186/s13019-023-02309-y.

## Introduction

Esophageal cancer (EC) is the sixth most common cancer and the eighth most common cause of cancer-related death [1]. The prognosis for EC patients is still poor, whose five-year overall survival (OS) rate is about 15–34% [2]. Radical esophagectomy with lymphadenectomy is still the dominant treatment for EC [3]. And most patients experience esophagogastric anastomosis because it reduces risk of anastomotic complications [4, 5]. Over the past few decades, thoracic surgeons have been improving esophagogastric anastomotic techniques to shorten the operation time and reduce the incidence of postoperative complications. However, the choice of anastomotic technique has been a controversial issue among different studies. The main basis for determining the technique is postoperative complications, such as anastomotic leakage, stricture and reflux esophagitis, which can affect the quality of life and even be life-threatening. The anastomotic leakage is critical to the OS of the patients and the anastomotic stricture can also directly affect the survival condition of patients. However, an optimal technique remains to be established in esophageal surgery that can promote the anastomosis healing and prevent the postoperative complications [6, 7].

Mechanical anastomosis, which consists of linear stapled (LS) anastomosis and circular stapled (CS) anastomosis, is the main technique used for clinical practice. Collard et al. [8] firstly showed the side-to-side anastomosis using a linear stapler. Then Orringer et al. [9] modified this technique in 2000. The CS anastomosis was found in 1990s, which has been a mature technique used today in the esophagogastric anastomosis. CS anastomosis and LS anastomosis have been widely accepted because they are less operator-dependent in comparison with the hand suture anastomosis.

Both CS and LS have their own merits and weaknesses. There have been many researches that aimed to compare CS and LS. However, there has been no agreement on which technique can reduce the postoperative complications. Zhou et al. [10] conducted a meta-analysis comparing CS with LS in 2015, which came to the conclusion that LS had a reduced rate of stricture, it should be noted that it has been seven years since this meta-analysis was published and the number of included studies was only five. Therefore, an up-to-date meta-analysis is needed to assess the many recent researches. We believe our analysis can be of some value for thoracic surgeons when faced with the choice between CS or LS anastomosis after esophagectomy. We present the following article in accordance with the PRISMA reporting checklist.

## Materials and methods

PRISMA [11] and MOOSE [12] guidelines were followed in our systematic review. Relevant papers in the database (Cochrane library, Web of science, Embase, PubMed) were searched systematically from establishment until March 2022 in our research. Relative medical subject heading terms, key words and word variants for “Esophagectomies” or “esophagectomy” or “esophagogastrostomy” or “esophagogastric” and “anastomosis” or “anastomotic” and “collard” or “linear” and “circular”. Other reports were finished by manual search of two authors in accordance with the reference lists of selected studies and reviews.

Whether the study had the eligibility for inclusion or not was determined by two observers in this meta-analysis. When inconsistencies were founded, another reviewer joined to discuss and reach an agreement. The same two observers also contributed to extracting data about study characteristics and outcome after getting the full text of included studies.

Studies were determined to include in the case where all the following criteria were met: (1) CS and LS anastomosis in esophagectomy were separated into groups; (2) comparative study; (3) the postoperative complications were recorded. Exclusion criteria was: (1) letters; (2) reviews or meta-analysis; (3) trials without CS vs. LS anastomosis.

The primary outcome in this study were classified into anastomotic leakage and stricture. The second outcome in this study were reflux esophagitis, pneumonia, the length of hospital stay and operation time. Patients were considered to fall into the primary outcome of anastomotic leakage when meeting the following indication: the contrast study was positive and the clinical signs were needed to alter in hospital stay, such as wound drainage or reoperation. If the patients experienced any dysphagia and needed several dilatation during the six months after the operation, we considered them to have the occurrence of stricture and postoperative dilatation was required. To support the diagnosis, anastomotic narrowing was noted when endoscopy and dysphagia were relieved after dilatation. This meta-analysis has been registered in the systematic review database of PROSPERO with registration number CRD42022324848.

### Statistical analyses

Review manager (version 5.4) software was used in this study. The pooled estimate was shown by forest plots. The odd ratio (OR) with 95% confidence interval (CI) was used to evaluate the dichotomous outcomes. The mean difference (MD) with 95% CI was used to present the continuous outcome varies. Hozo et al. [13] provided a method to assess the mean and standard deviation (SD) where the included studies merely have the report of medians and range. We used the Higgins I²statistics to assess the statistical heterogeneity between studies. If there was small heterogeneity (I²<50%), the fixed-effect model was used. Otherwise, the random-effect model would be used. P < 0.05 was considered statistically significant. Stratification by anastomotic location was performed to exclude the impact of anastomotic location on the outcome. The Review manager (version 5.4) software was used to evaluate the risk of bias, which contained seven items: random sequence generation, allocation concealment, blinding of participants and personnel, blinding of outcome assessment, incomplete outcome data, selective reporting, and other biases. The risk of bias assessment was carried out by two reviewers independently. A third reviewer arbitrated unresolved disagreements. The potential bias was graded as ‘high risk’, ‘low risk’ or ‘unclear risk’. Publication bias was assessed using funnel plot (Figure S1) (Figure S2).

## Results

### General characteristics

**Fig. 1:**
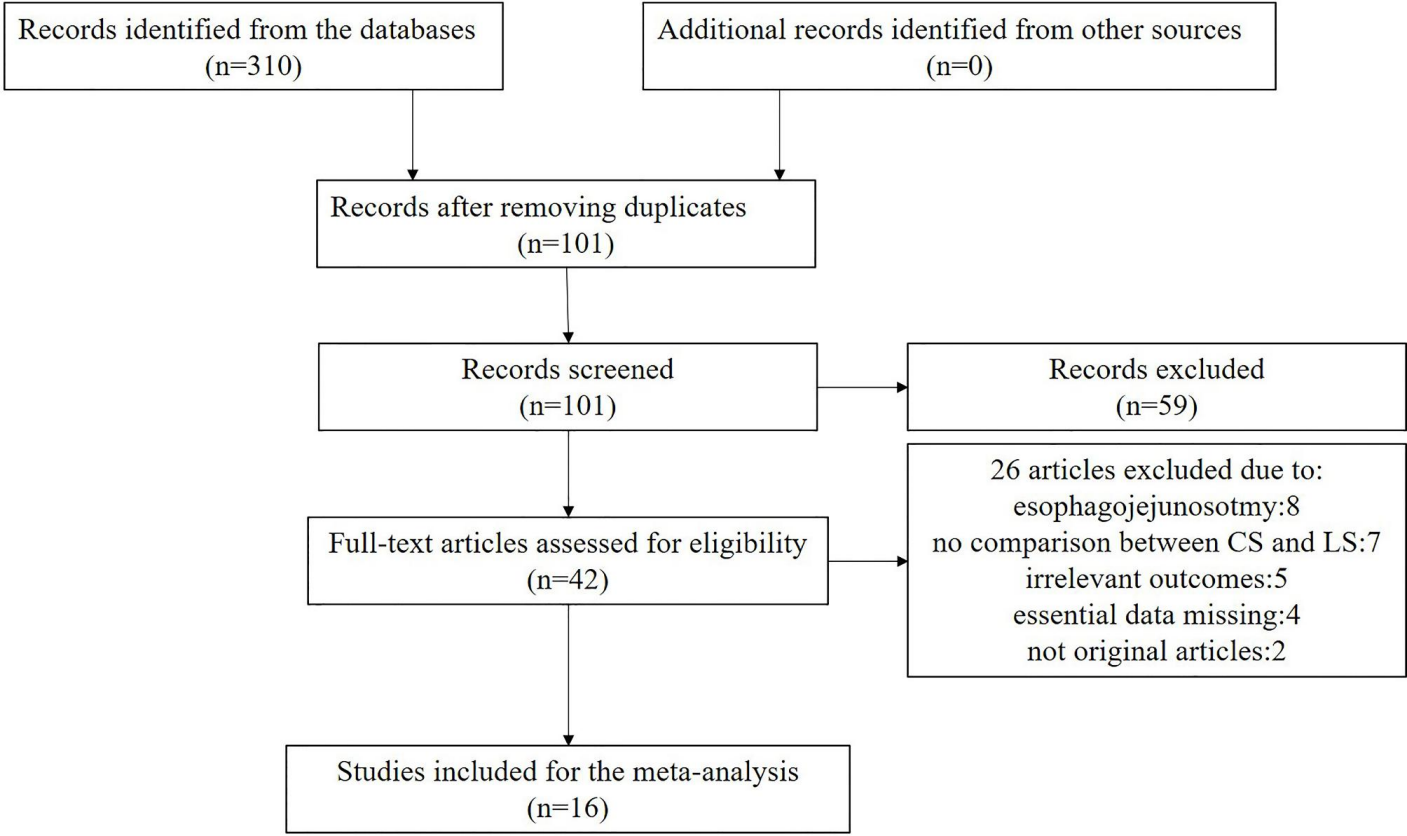
Flow chart of selecting for included studies

**Fig. 2:**
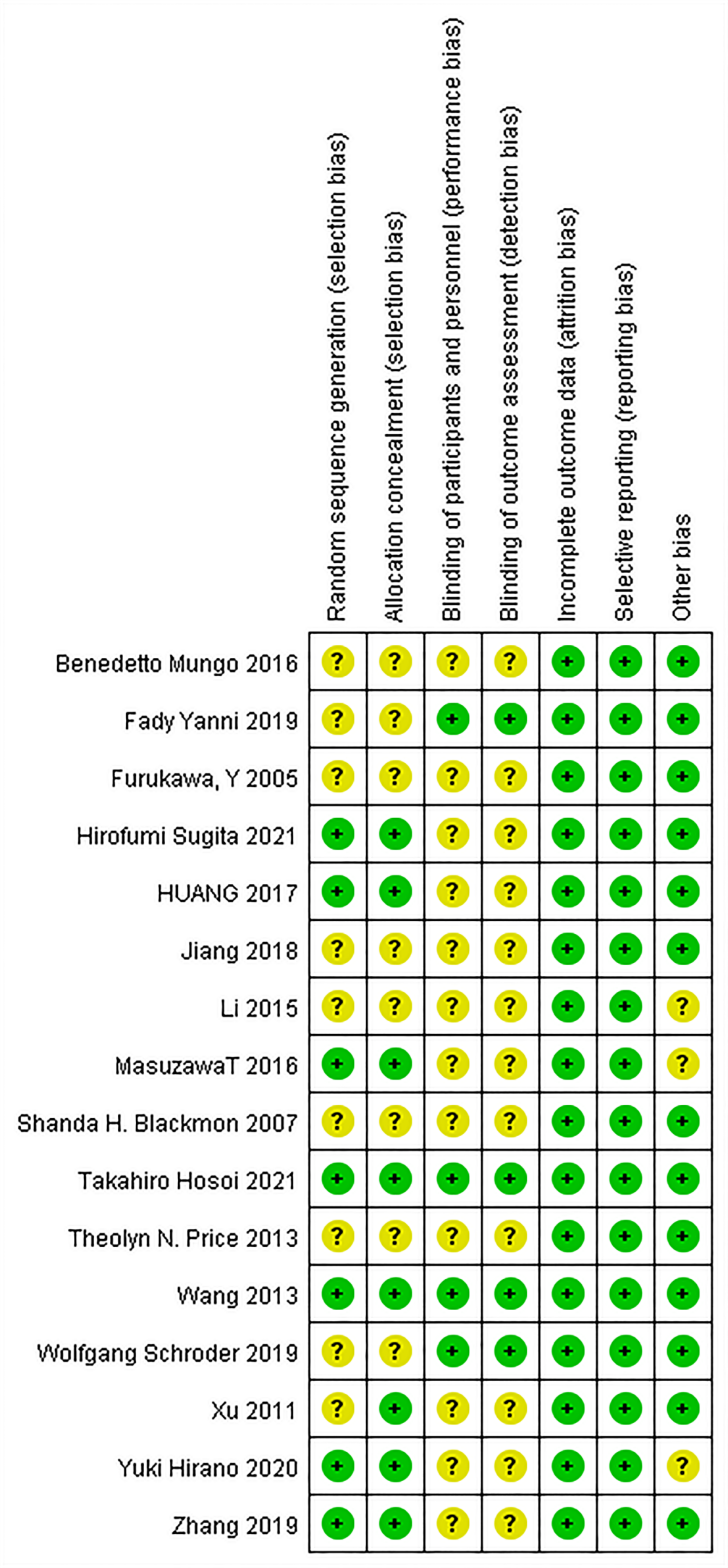
Risk of bias assessed by the judgement of authors

After finishing the screen, 16 studies with a total patient number of 2322 (1195 CS vs. 1127 LS) were included. Table 1 showed the patient demographic data. A flowchart (Fig. 1) showed the process of the literature search in the meta-analysis. Figure 2 showed the risk of bias assessment in the included studies, most of which were of moderate quality.

**Table 1:** Characteristics of 16 eligible studies † **LS**, linear stapled anastomosis; ‡ **CS**, circular stapled anastomosis; §**NA**, not available

Study	Year	Country	Study type	Site	Total		Leak		Stricture		AGE,years		BMI,kg/m²	
					CS	LS	CS	LS	CS	LS	CS	LS	CS	LS
*Hirofumi Sugita*	*2021*	*JAPAN*	retrospective	Thoracic	30	30	1	1	5	2	72[47–87]	72[51–87]	24.7[18.5–30.2]	24.7[18.5–30.2]
*Takahiro Hosoi*	*2021*	*JAPAN*	RCT	Cervical	42	53	3	4	18	0	70[46–81]	66[47–82]	21.8[15.3–29.7]	21.1[15.8–28.0]
*Yuki Hirano*	*2020*	*JAPAN*	retrospective	Cervical	86	86	10	11	24	12	66 ± 8	65 ± 8	21.4 ± 2.6	21.4 ± 3.6
*Zhang*	*2019*	*CHINA*	prospective	Thoracic	42	35	2	3	3	2	61.9 ± 7.8	61.4 ± 9.2	22.9 ± 2.2	22.6 ± 4.4
*Fady Yanni*	*2019*	*UK*	retrospective	Thoracic	85	74	13	3	8	4	68	65.5	NA	NA
*Wolfgang Schroder*	*2019*	*RUSSIA*	retrospective	Thoracic	427	109	135	14	NA	NA	64[57–70]	64[57–70]	26 [23–29]	26[23–29]
*Jiang*	*2018*	*CHINA*	prospective	Cervical	164	34	14	3	31	1	NA	NA	NA	NA
*Huang*	*2017*	*CHINA*	retrospective	Cervical	42	39	8	3	10	1	57.9 ± 8.2	61.0 ± 8.9	21.5 ± 3.5	22.8 ± 3.3
*MasuzawaT*	*2016*	*JAPAN*	retrospective	Cervical	31	12	5	1	6	1	NA	NA	NA	NA
*Benedetto Mungo*	*2016*	*USA*	prospective	Thoracic	38	12	4	2	NA	NA	66[63.5–72.5]	66[63.5–72.5]	26.9 (24.3, 26.9)	26.9 (24.3, 26.9)
*Li*	*2015*	*CHINA*	retrospective	MIXED	14	70	2	0	2	2	NA	NA	NA	NA
*Wang*	*2013*	*CHINA*	RCT	Thoracic	47	35	0	1	9	0	61.4 ± 7.7	59.7 ± 7.4	NA	NA
*Theolyn N.Price*	*2013*	*USA*	prospective	MIXED	48	327	4	35	15	49	NA	NA	NA	NA
*XU*	*2011*	*CHINA*	prospective	Thoracic	68	166	1	2	14	3	61.3 ± 7.6	60.2 ± 8.4	NA	NA
*Shanda H. Blackmon*	*2007*	*USA*	prospective	Cervical	23	23	1	2	2	2	62 ± 12	61 ± 9	26 ± 5	26 ± 4
*Furukawa Y*	*2005*	*JAPAN*	retrospective	NA	8	12	2	1	4	1	NA	NA	NA	NA

### Gastroesophageal anastomotic leakage

**Fig. 3:**
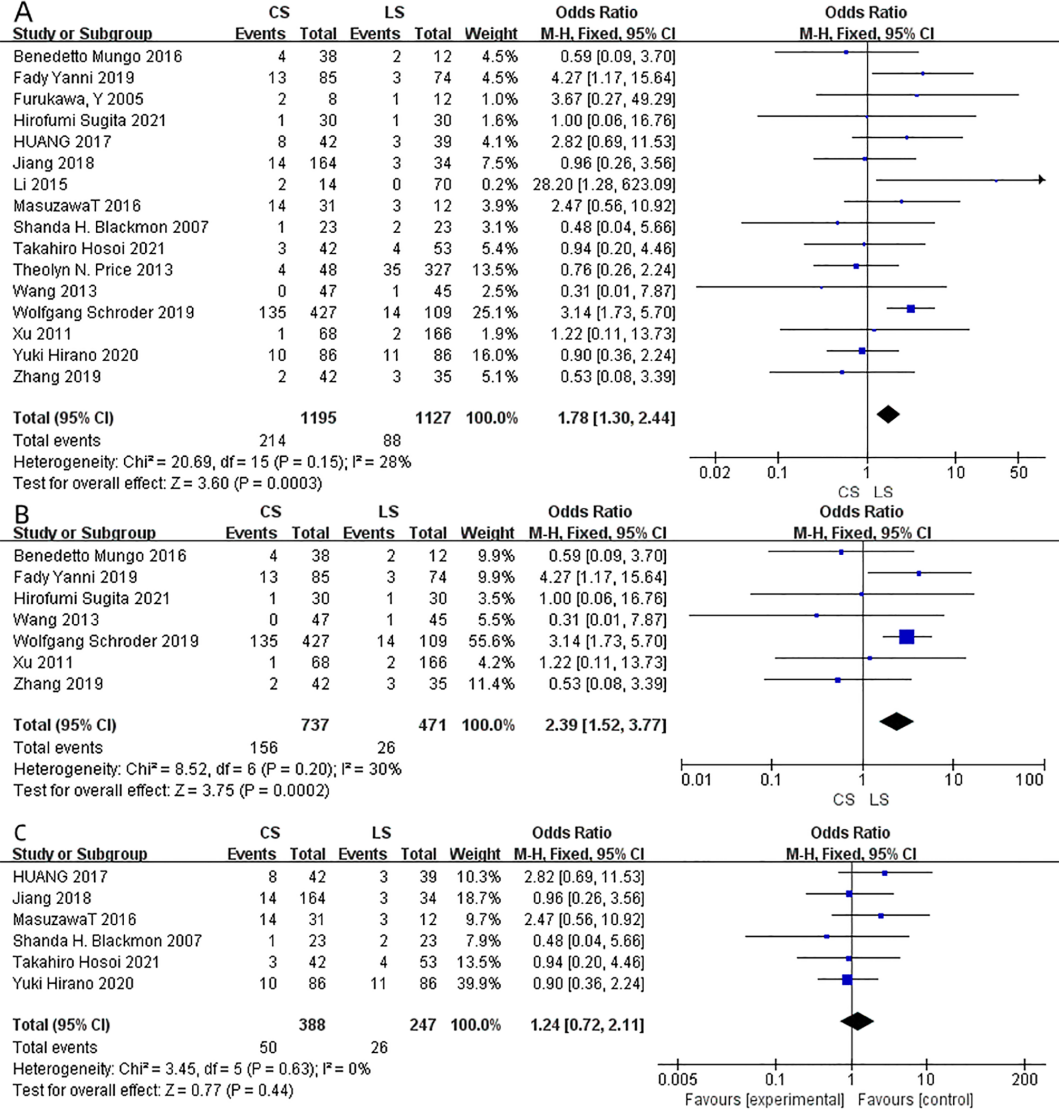
Forest plot on anastomotic leakage (**A**) anastomotic leakage in thoracic anastomosis (**B**) anastomotic leakage in cervical anastomosis (**C**) comparison between CS and LS.

Anastomotic leakage was reported in all studies including 2322 patients (1195 in CS group vs. 1127 in LS group). The risk of anastomotic leakage was significantly reduced in the use of LS anastomosis in comparison to the CS group (OR = 1.78; 95% CI = 1.30–2.44; I²=28%; P = 0.0003) (Fig. 3A). After being stratified by the anastomotic site, LS anastomosis was associated with a reduced risk of anastomotic leakage (OR = 2.39; 95% CI = 1.52–3.77; I²=30%; P = 0.0002) (Fig. 3B) in thoracic anastomosis. However, no significant difference was found in the cervical anastomosis (OR = 1.24; 95% CI = 0.72–2.11; I²=0; P = 0.44) (Fig. 3C).

### Gastroesophageal anastomotic stricture

**Fig. 4:**
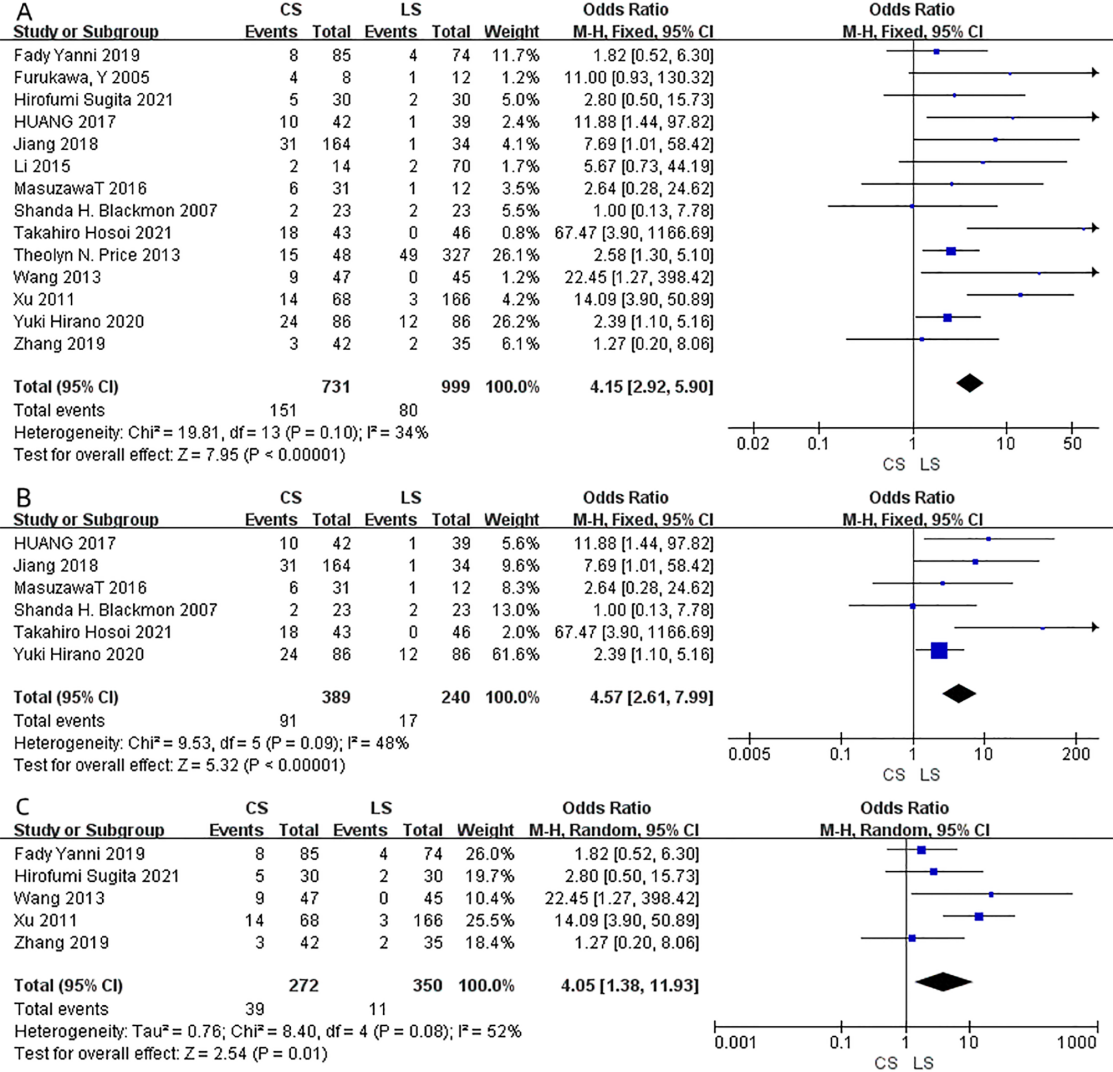
Forest plot on anastomotic stricture (**A**) anastomotic stricture in cervical anastomosis (**B**) anastomotic stricture in thoracic anastomosis (**C**) comparison between CS and LS

Anastomotic stricture was reported in fourteen studies including 1730 patients (731 in CS group vs. 999 in LS group). The LS anastomosis had significantly a reduced risk of the incidence of anastomotic stricture (OR = 4.15; 95% CI = 2.92–5.90; I²=34%; P < 0.00001) (Fig. 4A) compared to the CS group. A sensitivity analysis was also conducted, in which 1 study at a time was removed and the others analyzed to estimate whether the results could have been affected markedly by a single study. The combined OR of overall risk estimates were consistent and without apparent fluctuation, with a range from 3.61 (95% CI = 2.51–5.18) to 4.71 (95% CI = 3.11–7.13). After being stratified by the anastomotic site, LS anastomosis reduced the risk of anastomotic stricture both in cervical group (OR = 4.57; 95% CI = 2.61–7.99; I²=48%; P < 0.00001) (Fig. 4B) and thoracic group (OR = 4.05; 95% CI = 1.38–11.93; I²=52%; P = 0.01) (Fig. 4C). The random-effect model was used where the statistical heterogeneity was found.

### Other complications and outcomes

**Fig. 5:**
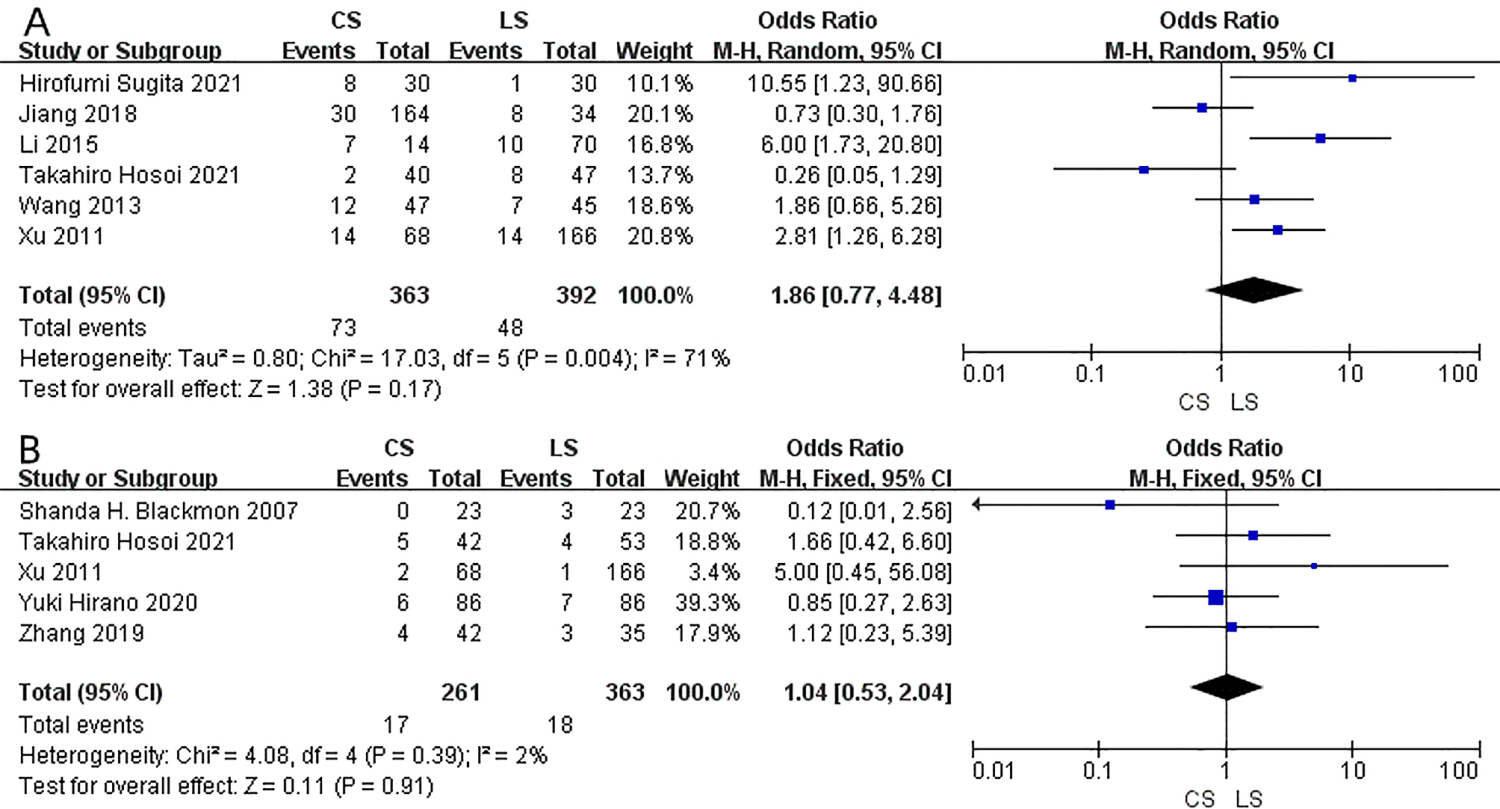
Forest plot of the reflux esophagitis (**A**) and pneumonia (**B**) comparison between CS and LS.

**Fig. 6:**
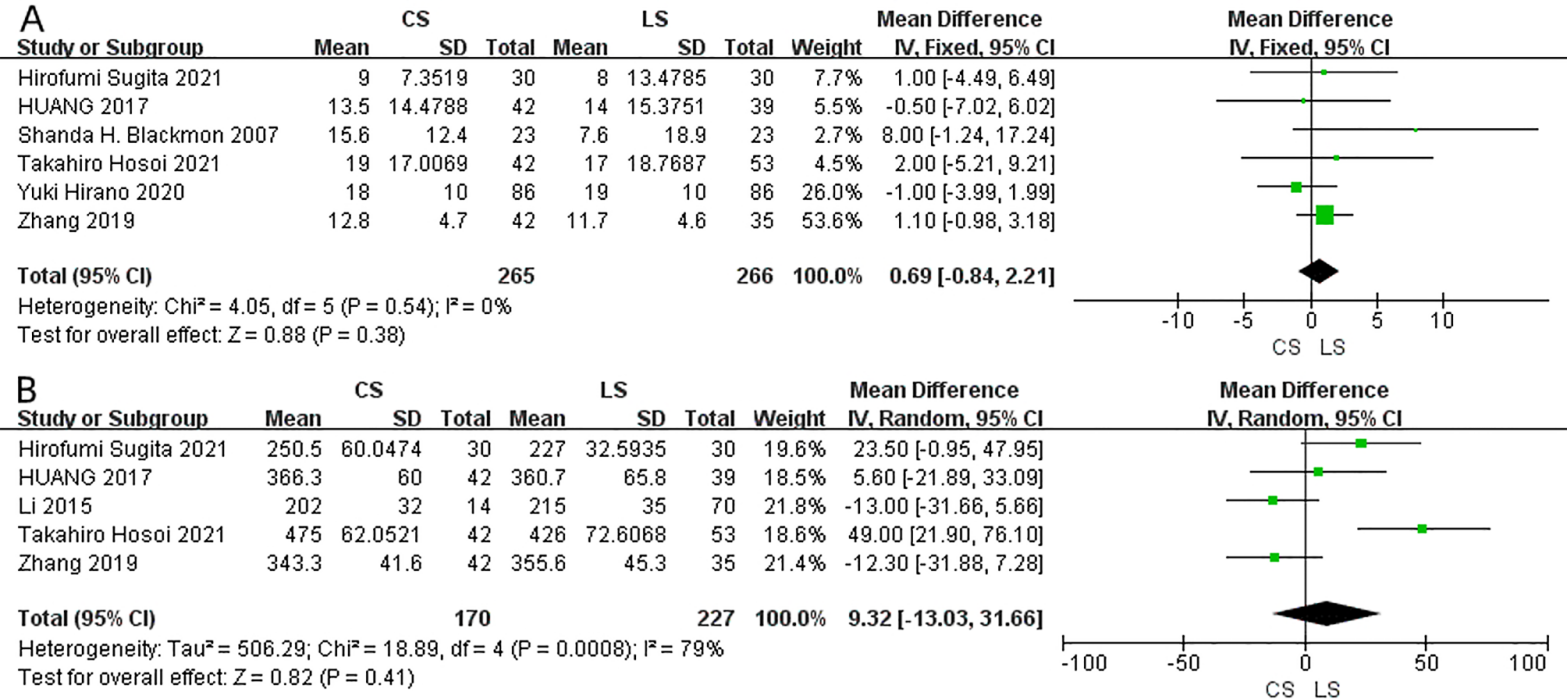
Forest plot of the length of hospital stay (**A**) and operation time (**B**) comparison between CS and LS.

Reflux esophagitis was reported in six studies. No significant difference between CS and LS anastomosis in the risk of reflux esophagitis was found (OR = 1.86; 95% CI = 0.77–4.48; I²=71%; P = 0.17) (Fig. 5A). The random-effect model was used because of the statistical heterogeneity. Pneumonia was reported in five studies. No significant difference was observed between the CS and LS anastomosis (OR = 1.04; 95% CI = 0.53–2.04; I²=2%; P = 0.91) (Fig. 5B). The length of hospital stay was reported in six studies, there was no significant difference between CS and LS anastomosis (MD = 0.69, 95% CI=-0.84-2.21; I²=0; P = 0.38) (Fig. 6A). Moreover, no significant difference was found in the operation time between CS and LS anastomosis (MD = 9.32; 95% CI=-13.03-31.66; I²=79%; P = 0.41) (Fig. 6B). The random-effect model was used where the statistical heterogeneity was found.

## Discussion

This study provided a comprehensive summation of recent literature showing the association of postoperative complications with LS or CS anastomosis. It demonstrated that the use of LS technique after esophagectomy could reduce the risk of both anastomotic leakage (P = 0.0003) and stricture (P < 0.00001) in comparison with CS technique. Stratification by anastomotic site showed that irrelevant of the anastomotic site (cervical or thoracic anastomosis), LS anastomosis could effectively reduce the anastomotic stricture in comparison with CS anastomosis. Moreover, LS anastomosis could significantly decrease the risk of thoracic anastomotic leakage. However, statistically significant difference was not found between CS and LS anastomosis in reflux esophagitis (P = 0.17), pneumonia (P = 0.91), operation time (P = 0.41) and the length of hospital stay (P = 0.38).

Anastomotic leakage is associated with long term cancer recurrence [14]. Previous studies have not reached agreement on whether CS or LS anastomosis can reduce the incidence of anastomotic leakage. Yanni et al. demonstrated a significant reduction in the risk of anastomotic leakage in using a LS anastomosis in comparison with CS anastomosis in thoracic anastomosis [15]. Huang et al. [16] also found LS anastomosis was effective and could be an anastomotic technique alteration after the esophagectomy because of the lower risk of anastomotic leakage. However, Hosoi et al. [17] reported that the association between the risk of anastomotic leakage and the two different anastomosis was comparable. Our study demonstrated that the LS anastomosis could reduce the risk of anastomotic leakage in comparison with the CS anastomosis, which was of major implication for thoracic surgeons. However, there is no difference in the occurrence of leakage in the cervical anastomosis, and we speculate that this may be related to other more important factors affecting anastomotic leakage, such as anastomotic tension and nutritional status. In future work, we will explore this issue further in patients with cervical anastomosis to further validate the findings of this study.

Anastomotic stricture is closely associated with patient morbidity and the need for further invasive procedures [18], which brings physical and psychological pain to patients. The anastomotic stricture rate using a circular stapler was 13.9–28.6% [19–22] and that using linear stapler was 0–20% [23–25]. The above data implies that anastomotic stricture was associated with the choice of CS anastomosis or LS anastomosis. Moreover, Xu et al. [26], Wang et al. [27], Theolyn N et al. [28], Mungo et al. [29], found the LS technique reduced the likelihood of anastomotic stricture compared with the CS anastomosis. In our study, the LS anastomosis was significantly demonstrated to reduce the risk of anastomotic stricture in comparison with CS anastomosis irrelevant of anastomotic site, which was consistent with previous studies.

Reflux esophagitis is still an unsolved problem which can really decrease life quality of patients after anastomosis. The reflux esophagitis rate after esophagectomy using CS anastomosis was 14.3–35.3% in previous studies and LS anastomosis was 30.0–30.8% [30, 31]. The above data seemed to show that LS anastomosis increased the risk of reflux esophagitis in comparison with the CS anastomosis. But some studies found the LS technique esophagogastrostomy could reduce the risk of anastomotic stricture without increasing the risk of gastroesophageal reflux [26, 27]. Our study indicates no significant difference between the CS and LS anastomosis. Our outcome was not very convincing because the analysis of reflux esophagitis involved was only six and the heterogeneity was large. From our perspective, the larger anastomotic width in LS anastomosis which prevents the incidence of intraluminal scarring may contribute to reducing reflux esophagitis. More RCTs are needed to explore the relationship between the choice of mechanical anastomotic technique and the incidence of reflux esophagitis.

In our study, the LS anastomosis has been established as the optimal technique in reducing the risk of postoperative complications in comparison with the CS anastomosis. From our clinical experience, there are several potential reasons. Firstly, CS technique has its drawbacks because the anastomotic lumen is needed to be matched with the corresponding esophageal width, conversely, the LS anastomosis is able to make a larger anastomotic lumen [32] and the anastomotic site could be enlarge by extension of the anastomosis along the posterior wall of the esophagus. Secondly, the use of LS anastomosis makes an extroverted anastomosis by using both sides of the lumen for anastomosis, which leads to exact mucosa-to-mucosa apposition. It effectively contributes to patient’s recovery. In contrast, the use of CS anastomosis makes an inverted anastomosis. Moreover, the CS anastomosis completes healing by hyperplasia of scar. The excessive scar probably leads to postoperative complications [33]. Last but not least, the enough blood supply to the distal anastomosis site after esophagogastric anastomosis is considered to reduce the risk of postoperative complications [34], and the use of CS anastomosis blocks the vascular network in stomach wall. Conversely, the use of LS anastomosis can preserve the vascular network of stomach wall to the utmost extent.

Meanwhile, the LS anastomosis has some limitations in that a longer remnant esophagus is needed in the LS anastomosis [17], especially in a case where esophageal tumor is on the top of the chest or neck. It is impossible to leave a normal esophageal tissue long enough in LS anastomosis to ensure the incision margin. Moreover, when the esophageal lumen is found to be clearly dilated during the operation, the LS technique is not necessary to enlarge the anastomotic site further. Therefore, thoracic surgeons should also work to develop novel anastomotic methods or technique to make the anastomosis operation safer.

However, there are some limitations in our study. Most studies in our research were not RCTs, so there may exist some bias. There were remained unexplained heterogeneity when exploring the reflux esophagitis, operation time and the stratified analysis in thoracic anastomotic stricture. The number of patients was relatively small in exploring the pneumonia, reflux esophagitis, operation time and the length of hospital stay. Moreover, the different technical levels of the surgeons may have a certain impact on the results. Despite these weakness, our meta-analysis represents the latest and most comprehensive published assessment of CS in comparison with LS in postoperative complications.

## Conclusions

The study demonstrated that the use of LS technique after esophagectomy could reduce the risk of both anastomotic leakage (P = 0.0003) and stricture (P < 0.00001) in comparison with CS technique. Stratification by anastomotic site showed that irrelevant of anastomotic site (cervical or thoracic anastomosis), LS anastomosis could effectively reduce the anastomotic stricture in comparison with CS anastomosis. Moreover, LS anastomosis could reduce the risk of thoracic anastomotic leakage. Therefore, the study suggested that LS could be considered to be an optimal treatment associated with reduced risk of anastomotic leakage and stricture in comparison with CS.

### Electronic supplementary material

Below is the link to the electronic supplementary material.

**Additional File Fig. S1.** The publishing bias in meta-analysis of the risk of anastomotic leakage between CS and LS

**Additional File Fig. S2.** The publishing bias in meta-analysis of the incidence of anastomotic stricture between CS and LS

**Additional File 3:** PRISMA 2020 Checklist

## Data Availability

Please contact author for data requests.

## Abbreviations

CI: Confidence interval
CS: Circular stapled
EC: Esophageal cancer
LS: Linear stapled
MD: Mean difference
OR: Odd ratio
OS: Overall survival
SD: Standard deviation
